# The 3/4- and 3/6-Subfamily Variants of α-Conotoxins GI and MI Exhibit Potent Inhibitory Activity against Muscular Nicotinic Acetylcholine Receptors

**DOI:** 10.3390/md19120705

**Published:** 2021-12-14

**Authors:** Xiaoli Ma, Qiuyuan Huang, Shuo Yu, Shujing Xu, Yue Huang, Zhiming Zhao, Xinrong Xiao, Qiuyun Dai

**Affiliations:** 1 Beijing Institute of Biotechnology, Beijing 100071, China; maxiaoli775621363@163.com (X.M.); huangqy654678@163.com (Q.H.); o_yys@163.com (S.Y.); xushujing0225@163.com (S.X.); vayuuuu@163.com (Y.H.); 13679460257@163.com (Z.Z.); 2 Institute of Chemistry and Chemical Engineering, University of South China, Henyang 421000, China

**Keywords:** α-conotoxins GI and MI, muscular nicotinic acetylcholine receptors, subfamily variants, structure–activity relationship

## Abstract

α-Conotoxins GI and MI belong to the 3/5 subfamily of α-conotoxins and potently inhibit muscular nicotinic acetylcholine receptors (nAChRs). To date, no 3/4- or 3/6-subfamily α-conotoxins have been reported to inhibit muscular nAChRs. In the present study, a series of new 3/4-, 3/6-, and 3/7-subfamily GI and MI variants were synthesized and functionally characterized by modifications of loop2. The results show that the 3/4-subfamily GI variant GI[∆8G]-II and the 3/6-subfamily variants GI[+13A], GI[+13R], and GI[+13K] displayed potent inhibition of muscular nAChRs expressed in *Xenopus* oocytes, with an IC_50_ of 45.4–73.4 nM, similar to or slightly lower than that of wild-type GI (42.0 nM). The toxicity of these GI variants in mice appeared to be about a half to a quarter of that of wild-type GI. At the same time, the 3/7-subfamily GI variants showed significantly lower in vitro potency and toxicity. On the other hand, similar to the 3/6-subfamily GI variants, the 3/6-subfamily MI variants MI[+14R] and MI[+14K] were also active after the addition of a basic amino acid, Arg or Lys, in loop2, but the activity was not maintained for the 3/4-subfamily MI variant MI[∆9G]. Interestingly, the disulfide bond connectivity “C1–C4, C2–C3” in the 3/4-subfamily variant GI[∆8G]-II was significantly more potent than the “C1–C3, C2–C4” connectivity found in wild-type GI and MI, suggesting that disulfide bond connectivity is easily affected in the rigid 3/4-subfamily α-conotoxins and that the disulfide bonds significantly impact the variants’ function. This work is the first to demonstrate that 3/4- and 3/6-subfamily α-conotoxins potently inhibit muscular nAChRs, expanding our knowledge of α-conotoxins and providing new motifs for their further modifications.

## 1. Introduction

Nicotinic acetylcholine receptors (nAChRs) are a class of ligand-gated ion channel protein receptors that mediate rapid signal transmission between synapses [1,2]. They are widely distributed in the central and peripheral nervous systems and muscles [3]. nAChRs are divided into two types: neuronal type and muscular type [3,4]. Neuronal nAChRs contain multiple different subunits, namely, α2–α7, α9, α10, and β2–β4, which constitute homopentamers or heteropentamers. Muscular nAChRs are composed of α1, β1, γ, δ, or ε subunits, and different subtypes of nAChRs mediate different pathophysiological functions [5]. Neuronal nAChRs are associated with a series of central nervous system dysfunctions, including pain, addiction, epilepsy, Alzheimer’s disease, Parkinson’s disease, and schizophrenia [6,7,8], while muscular nAChRs are associated with physiological functions (such as muscular movement and signal transmission) and pathophysiological conditions [1,5], including pain, myogenic diseases, muscular dystrophy, and myasthenia gravis.

α-Conotoxins are small peptides composed of 12–20 amino acids, containing two pairs of disulfide bonds with a main globular disulfide bond framework “C1–C3, C2–C4” [9,10,11]. The general structural formula of α-conotoxins is –CC–(loop 1)–C–(loop 2)–C– [1]. According to the number of residues in loop 1 and loop 2, α-conotoxins can be further divided into several structural subfamilies such as 3/5, 4/3, 4/4, 4/5, 4/6, and 4/7 subfamilies [12,13,14]. They are potent antagonists of nAChRs, and studies on structure–activity relationship have shown that the loop1 of α-conotoxins plays a key role in the selectivity of nAChR types [9,13] and the number of amino acids in loop1 has little effect on loop1 structure. On the other hand, the loop2 of α-conotoxins plays a key role in the inhibitory activity and selectivity of nAChR subtypes [11,15]. In general, the 3/5 subfamily of α-conotoxins mainly target fish or mammalian neuromuscular nAChRs; typical toxin members include GI [16,17,18,19], MI [20,21], GII [22], GIA [22], SIA [23], SI [24,25], CnIA [26], CIA [27], CIB [27], and MilIA [28]. A number of other conotoxins have also been found to act on neuromuscular nAChRs, such as EI [29], PIVA [30], OIVA [31], OIVB [32], PIB [33], EIVA [34], EIIA [35], PIC [36], and MIIIJ [37]. On the other hand, 4/3, 4/4, 4/5, 4/6, and 4/7 α-conotoxins usually target neuronal nAChRs [11,13]. Among the conotoxins reported, GI and MI are the most potent. To date, no 3/4-, 3/6-, and 3/7-subfamily α-conotoxins have been reported.

In order to discover new subfamilies of α-conotoxins targeting muscular nAChRs and provide new pharmacological probe molecules, in the present study, a series of 3/4-, 3/6-, and 3/7-subfamily variants of α-conotoxins GI and MI were synthesized, and their inhibitory activities against muscular nAChRs and toxicity in mice were determined. Surprisingly, it was found that with the deletion of the first amino acid Gly in loop2 of GI, the generated linear peptide variant, GI[∆8G]-II, mainly folded with the disulfide bond connection “C1–C4, C2–C3” instead of the wild-type “C1–C3, C2–C4” connection. The 3/4-subfamily variant GI[∆8G]-II and the 3/6-subfamily variants GI[+13A], GI[+13R], GI[+13K] displayed high potency in inhibiting muscular nAChRs, with a IC_50_ of 45.4–73.4 nM, comparable to or slightly lower than that of wild-type GI (42.0 nM), while the toxicity in mice was about a half to a quarter of that of wild-type GI. The 3/7-subfamily GI variants showed significantly lower potency in vitro and toxicity in vivo compared to wild-type GI. In addition, 3/4-, 3/6-, and 3/7-subfamily MI variants were investigated, and the on-rate and off-rate kinetics of inhibition of muscular nAChRs by GI, MI, and their variants were also determined. This work is the first to identify the inhibitory functions of 3/4- and 3/6-subfamily α-conotoxins towards muscular nAChRs. This expands our knowledge of α-conotoxins and provides new motifs for their further modifications.

## 2. Results

### 2.1. Synthesis and Characterization of Conotoxins

**Figure 1:**
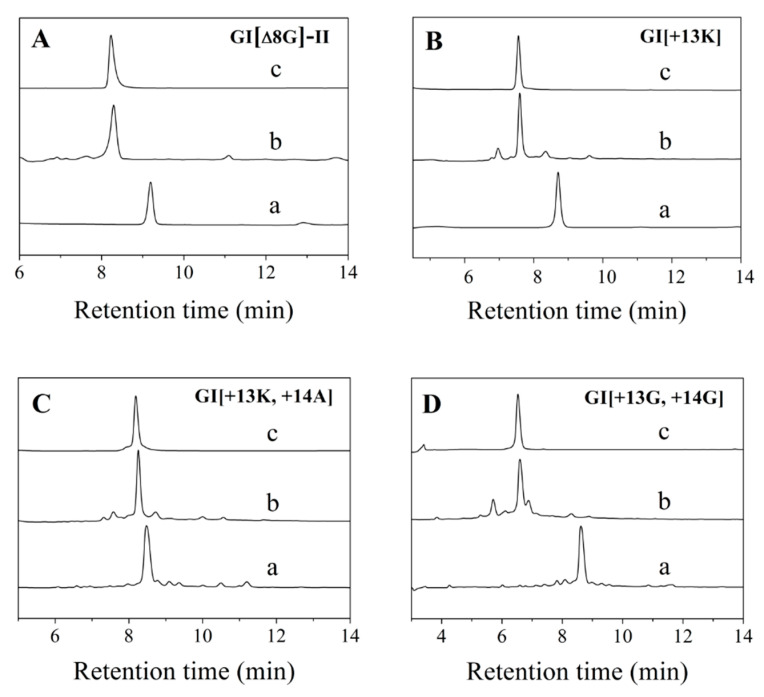
HPLC analyses of one-step folding products of linear GI variants. (**A**) GI[ΔG8]-II. (**B**) GI[+13K]. (**C**) GI[+13K, +14A]. (**D**) GI[+13G, +14G].Traces from bottom to top: linear peptide (a), folded products (b), purified products (c). Samples were applied to an Agilent Eclipse Plus C18 column (5 μm, 4.6 mm×250 mm) and eluted with a linear gradient of 5–10% B for 0–1 min; 10–50% B (B is acetonitrile containing 0.1% trifluoroacetic acid (TFA)) for 1–25 min. Absorbance was monitored at 214 nm. Flow rate was 1.0 mL/min.

A series of 3/4-, 3/6-, and 3/7-subfamily variants of α-conotoxins GI and MI were designed and synthesized (Table 1). Linear GI, MI, and their variants were folded individually in 0.1 mM ammonium acetate buffer and analyzed by HPLC. The analytical results of the typical folding products of GI[Δ8G]-II, GI[+13K], GI[+13K, +14A] and GI[+13G, +14G] are shown in Figure 1. All variants showed a main peak, and the purity was >95% after purification. Their molecular weight was determined by ProFLEXTM-III MALDI-TOF mass spectrometry and was consistent with the theoretical molecular weight (Table 1).

### 2.2. Disulfide Bond Connectivity

**Figure 2:**
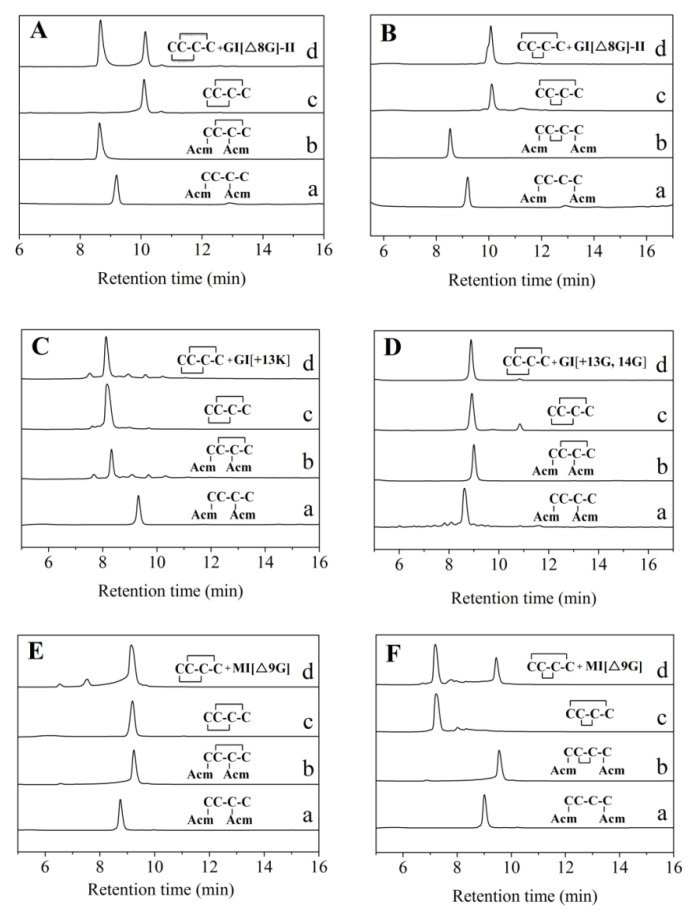
HPLC analyses of the folded products of linear GI and MI variants with Acm modification. (**A**) Linear GI[Δ8G]-I with Acm modifications at Cys1 and Cys3. (**B**) Linear GI[Δ8G]-II with Acm modifications at Cys1 and Cys4. (**C**) Linear GI[+13K] with Acm modifications at Cys1 and Cys3. (**D**) Linear GI[+13G, +14G] with Acm modifications at Cys1 and Cys3. (**E**) Linear MI[Δ9G]-I with Acm modifications at Cys1 and Cys3. (**F**) Linear MI[Δ9G]-II with Acm modifications at Cys1 and Cys4. Traces from bottom to top: linear peptide (a), primary oxidized product (b), secondary oxidized product (c), and co-elution of the two-step folding product and one-step natural-air folding oxidation product (d). HPLC analysis conditions are the same as in Figure 1.

The first folding of the GI[Δ8G]-I[EC(Acm)CNPAC(Acm)RHYSC] linear peptide modified by Acm at position 1,3 formed a product containing a “C2–C4” disulfide bond (Figure 2A). After the Acm protecting group was removed by iodine oxidation, the main product containing disulfide bond connectivity “C1–C3, C2–C4” formed (Figure 2A(c)). On the other hand, for GI[Δ8G]-II, the mixture of the two-step folding product (GI[Δ8G]-I) and the one-step folding product showed two peaks, indicating the disulfide bond connectivity of the one-step folding product was not “C1–C3, C2–C4”. The final folded product of Acm-modified GI[Δ8G]-II at “C1, C4” showed a single peak when it was mixed with the one-step folding product (Figure 2B), indicating its disulfide bond connectivity was “C1–C4, C2–C3”. Using a similar method, the disulfide bond connectivity of the one-step folding products of GI[+13K], GI[+13G, +14G], and MI[Δ9G] were also determined. The results demonstrated that these variants have the same disulfide bond connectivity “C1–C3, C2–C4” as those of wild-type GI and MI (Figure 2C–F).

### 2.3. Circular Dichroism (CD) Spectroscopy

**Figure 3:**
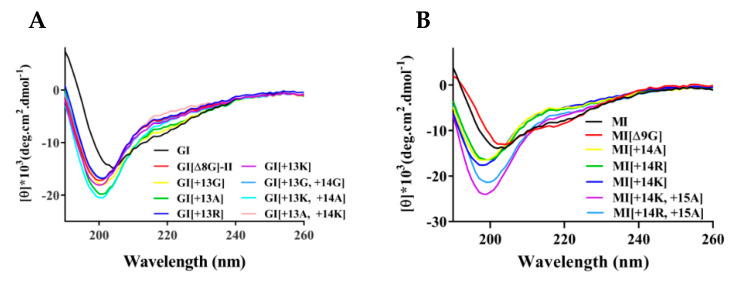
Circular dichroism spectra of α-conotoxins and their variants in 0.01 M PBS (pH = 7.2). (**A**) GI mutants. (**B**) MI mutants. *n* = 3.

CD spectra (Figure 3) of the GI and MI variants showed that wild-type GI and MI have a certain number of α-helices because they exhibit negative peaks at 222 and 208 nm, consistent with their NMR structures [38,39]. However, after the addition of one or two amino acids in loop 2, the α-helix content of the variants, such as GI[+13R], GI[+13K], GI[+13A], GI[+13K, +14A], GI[+13G, +14G], MI[+14R], MI[+14K], MI[+14K, +15A], was significantly reduced. Some variants only exhibited a negative peak near 198 nm, indicating a random coil structure. GI[ΔG8]-II appeared to have almost a random coil structure because its disulfide bond connectivity was “1–4, 2–3”, but the CD spectrum of MI[Δ9G] was similar to that of MI.

### 2.4. Inhibitory Activity of α-Conotoxins GI and MI Variants against Muscular nAChRs

**Figure 4:**
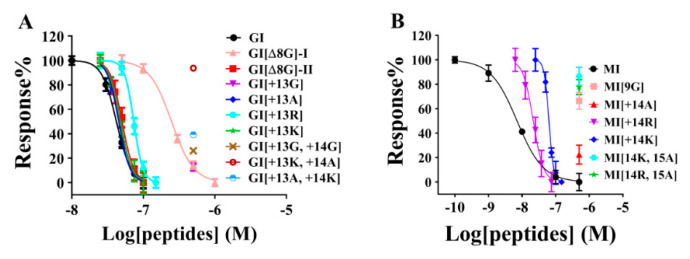
Concentration-dependent response curves of the GI and MI variants interacting with rat muscular nAChRs. (**A**) GI variants; (**B**) MI variants. The error bars for the data denote the SEM. Five to seven oocytes were used for each determination. The IC_50_ values and 95% confidence intervals are summarized in Table 1.

The inhibitory activity of GI and MI variants toward muscular nAChRs expressed in *Xenopus* oocytes was determined. The results showed that wild-type GI exhibited a strong inhibition of the rat muscular nAChR, with an IC_50_ of 42.0 nM (Figure 4 and Table 1). After the deletion of Gly in loop2, the inhibitory activity of GI[∆8G]-II decreased slightly (IC_50_ = 50.4 nM), which is in contrast with what observed for the disulfide bond isomer GI[∆8G]-I: the latter displayed lower potency (IC_50_ = 248.1 nM), nearly a four-fold decrease compared to the wild-type. After the addition of one amino acid residue in loop 2 (such as Ala, Lys, and Arg), the IC_50_ values changed to 45.4 nM, 47.5 nM, and 73.5 nM for the 3/6-subfamily variants GI[+13A], GI[+13K], and GI[+13R], respectively, comparable to or slightly lower than the values of wild-type GI at 42.0 nM. However, the addition of two residues (GG or KA or AK) in loop2 resulted in a sharp decrease in potency (IC_50_ > 200 nM). Similar modifications were performed for MI. The results showed that wild-type MI potently inhibited the muscle-type nAChRs, with an IC_50_ of 7.0 nM, but differently from what observed for GI[∆8G], with the deletion of Gly9 in loop2, MI[∆9G] became inactive (IC_50_ > 500 nM), despite possessing the same disulfide bond connectivity as wild-type MI. Similar to the GI variants, the 3/6-subfamily MI[+14R] and MI[+14K] variants were also active after the addition of the basic amino acids Arg or Lys at loop2, while the 3/7-subfamily variants of MI lacked inhibitory activity (IC_50_ > 500 nM).

### 2.5. On-Rate and Off-Rate Kinetics for α-Conotoxins GI and MI Variants Interacting with Muscular nAChRs

**Figure 5:**
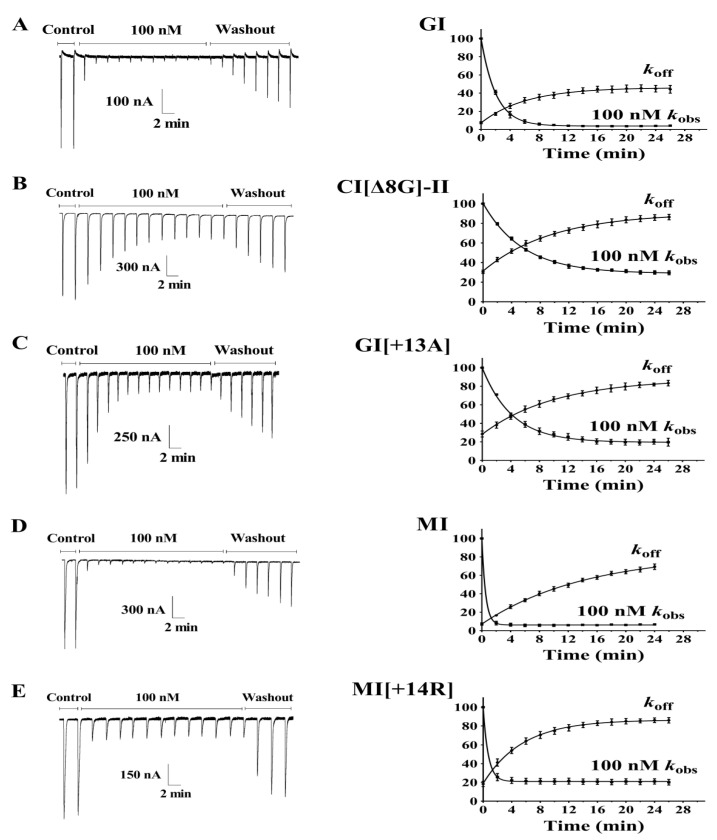
Kinetic analyses of GI and MI variants interacting with *Xenopus* oocyte-expressed rat muscular nAChRs. (**A**) GI, (**B**) GI[∆8G], (**C**) GI[+13A] (**D**) MI, (**E**) MI[+14R]. The toxins were applied as described in Materials and Methods, and the data were fit to a single exponential equation. The error bars denote the SEM of the data obtained using four to seven oocytes for each determination. The kinetic data are summarized in Table 2.

The on-rate and off-rate kinetics of GI, MI, and their variants with a high potency interacting with muscular nAChRs were determined (Figure 5, Table 2). After wild-type GI (100 nM) bound to muscular nAChRs, the time for 50% recovery (*t*_1/2_) of nAChRs was 4.26 min. Compared to wild-type GI, the dissociation rates of GI[∆8G]-II and GI[+13A] were slower, with *t*_1/2_ at 6.63 min and 7.04 min, respectively, but the corresponding recovery rates of GI[∆8G]-II (85.9%) and GI[+13A] (81.9%) were significantly higher than that of GI (44.9%) at 24 min. Accordingly, the inhibition constants (*K_i_*) of GI[∆8G]-II and GI[+13A] were 125.0 M^−9^ and 76.92 M^−9^, lower than that of wild-type GI (48.48 M^−9^). The addition of amino acids in MI resulted in a similar behavior of MI[+14R], which had a higher inhibition constant *Ki* compared to wild-type MI, but more easily dissociated from nAChRs, and displayed a lower *t*_1/2_ (3.78 min) than wild-type MI (*t*_1/2_ = 9.82 min). These results demonstrated that GI[∆8G]-II, GI[+13A], and MI[+14R] dissociate more easily from nAChRs compared to their wild-type counterparts.

### 2.6. Toxicity of GI and MI Variants in Mice

The toxicity of GI and MI variants was determined in mice (Table 3). The doses of wild-type GI and MI were 40 and 20 µg/kg, which resulted in 90–100% mortality in mice. The 3/4-subfamily GI variant GI[Δ8G]-II exhibited medium toxicity, with dose levels of 80, 120, 160 µg/kg leading to death rates of 0, 60, and 80%, which were around four-fold lower than that of GI at the corresponding dose. After the addition of one basic amino acid residue (such as Arg and Lys) in loop2 of GI, the toxicity did not decrease as dramatically. The doses of 80 and 120 µg/kg of GI[+13R] and GI[+13K] resulted in a death rate of 80–100%, which indicated a toxicity around two-fold lower than that of wild-type GI. In contrast, adding alanine and glycine to loop2 significantly decreased the toxicity, with 120 µg/kg or higher of GI[+13A] and GI[+13G] required to induce obvious death in mice. The 3/7-subfamily GI variants generally displayed low toxicity: GI[+13A, +14K] exhibited similar toxicity to GI[+13G], while the toxicity of GI[+13K, +14A] and GI[+13G, +14G] were almost lost (200 µg/kg resulted in no death).

For the MI variants, the 3/4-, 3/6-, and 3/7-subfamily MI variants displayed dramatically decreased toxicity, with the most toxic MI[+14K] leading to a mortality rate of only 20% at a high dose of 200 µg/kg, while all other MI variants did not induce any death even at 200 µg/kg.

## 3. Discussion

In the present work, we synthesized a series of variants of α-conotoxins GI and MI and identified their inhibitory activity against muscular nAChRs as well as their toxicity in mice. Through the deletion of a Gly residue in loop2, we obtained a 3/4-subfamily α-conotoxin GI[Δ8G]-II which displayed 80% of wild-type GI’s inhibitory activity against muscular nAChRs. In addition, GI[Δ8G]-II exhibited medium toxicity, as 160 µg/kg could result in 80% mortality in mice (Table 3). GI[Δ8G]-II is the shortest α-conotoxin reported to date and it is the first reported 3/4-subfamily α-conotoxin that can potently inhibit muscular nAChRs. Moreover, we found that the 3/6-subfamily α-conotoxins GI[+13A], GI[+13K], and GI[+13R] displayed similar potency toward muscular nAChRs as wild-type GI. This is also the first report of 3/6-subfamily α-conotoxins with high inhibitory activity toward muscular nAChRs and high toxicity.

A surprising finding of this report is that the linear peptide GI[Δ8G], formed after deletion of a Gly residue in loop2 of wild-type GI, forms the disulfide bond connectivity “1–4, 2–3” instead of the disulfide bond connectivity “1–3, 2–4” found in wild-type GI. Furthermore, the disulfide bond isomer (GI[Δ8G]-II) exhibited four-fold higher potency than the disulfide bond isomer (GI[Δ8G]-I) (Table 1). This phenomenon is unique in α-conotoxins targeting muscular nAChRs [40] and did not occur for the 3/4-subfamily MI variant and wild-type GI or MI. The linear peptide MI[Δ9G] forms a disulfide bond connectivity “1–3, 2–4” (the same as that of wild-type MI) and displayed low potency. We also determined the disulfide bond connectivity of the 3/6-, 3/7-subfamily GI and MI variants and found that they possess the disulfide bond connectivity “C1–C3, C2–C4”, the same as in wild-type GI and MI. These results suggest that the disulfide bond connectivity is easily affected in the rigid 3/4-subfamily α-conotoxins and that the disulfide bonds significantly impact variants’ function.

It was also found that the extension of loop2 of GI and MI is limited to six amino acids. With six amino acids, the 3/6-subfamily variants exhibited obvious inhibitory activity, and the addition of the basic amino acid Arg or Lys in loop2 could further elevate their potency. However, seven amino acids resulted in loss of potency. despite the addition of basic amino acids, as demonstrated by the inhibitory activities of the 3/7-subfamily variants GI[+13G, +14G], GI[+13K, +14A], GI[+13A, +14K], MI[+14K, +15A], and MI[+14R, +15A]. This could be attributed to an increase in flexibility, which resulted in a decreased binding of α-conotoxin to nAChRs. Correspondingly, the CD spectra also showed that a random coil structure exists in the 3/7-subfamily α-conotoxins.

It should be noted that the toxicity of the GI and MI variants did not completely correspond to their inhibitory activity. For example, the 3/4- and 3/6-subfamily GI variants GI[∆8G], GI[+13A], and GI[+13K] showed similar inhibitory activities as wild-type GI (Figure 4, Table 1), and the 3/6-subfamily MI[+14R] appeared to be even more active than GI (IC_50_ = 22.2 nM) (Table 1), but their toxicities were significantly lower that of wild-type GI. In fact, 80 µg/kg or a higher dose was required to produce obvious death in mice, a two-fold lower potency than expected. In order to interpret these results, we performed kinetics analyses. The results showed that the recovery rates of GI[∆8G]-II and GI[+13A] were significantly higher than that of GI (Table 2), which may explain the low toxicity. For MI[+14R], the on-rate and off-rate kinetics could not explain its low toxicity. The binding sites of the GI and MI variants may be partially different from that of wild-type GI or MI.

In summary, we produced 3/4-subfamily α-conotoxins with the disulfide bond connectivity “C1–C4, C2–C3”and a series of 3/6-subfamily α-conotoxins by the modifications of loop2 of GI and MI. They displayed potent inhibitory activities toward muscular nAChRs and low toxicity in mice. These shortest and larger α-conotoxins, as regards loop2, expand our knowledge of α-conotoxins, providing new motifs for further modifications of α-conotoxins.

## 4. Materials and Methods

### 4.1. Materials, Reagents, and Animals

Rink resin was obtained from Tianjin Nankai Hecheng S&T Company. N-Fmoc-amino acids, 1-hydroxybenzotriazole (HOBt), 2-(1H-benzotriazole-1-yl)-1,1,3,3-tetramethyluronium hexafluorophosphate (HBTU), *N,N*-diisopropyl ethylamine (DIEA), and TFA were purchased from GL Biochem Ltd. (Shanghai, China). DTT was obtained from Beijing Yinuokai Technology Co., Ltd. (Beijing, China). TIPS; TFA was purchased from Beijing Coupling Technology Co., Ltd. (Beijing, China). Iodine and ascorbic acid were purchased from Chemical Reagent Co., and Ltd. (Beijing, China). Acetonitrile was from fisher chemical. Muscular nAChRs receptor plasmids were from Addgene Company (Watertown, MA, USA).

Kunming mice (18–22 g, 3–4 weeks old) were obtained from SPF (Beijing, China) Biotechnology Co., Ltd., and housed at 23 ± 2 °C with a relative humidity of 50% under a 12 h light/dark cycle. Food pellets and water were available ad libitum. Female *Xenopus laevis* frogs (Cat. No. 20170127, 100–150 g, 24–36 months old) were obtained from the Research Center for Eco-Environmental Sciences (Beijing, China). All experiments were conducted in accordance with the guidelines of the Animal Research Advisory Committee of Beijing Institutes for Biological Science and conformed to the European Community directives for the care and use of laboratory animals.

### 4.2. Peptide Synthesis and Folding

Peptides with protecting groups were synthesized using a Sophas peptide synthesizer (Zinsser Analytic, Frankfurt, Germany) by the Standard Fmoc method and then cleaved from the Rink resin (0.05 mmol) in a mixed solution (4.4 mL TFA, 0.25 mL water, 0.25 g DTT, 0.1 mL triisopropylsilane). The resulting linear peptide was folded in 0.1 M ammonium acetate (pH 8.0–8.20) for 24–48 h. The folding progress was detected by C18 HPLC. After the folding was completed, the pH was adjusted to 4–5 with acetic acid to terminate the reaction, and then the folding liquid was desalted and loaded on a Nucleosil 25 × 250 mm preparative C18 column using a preparative HPLC pump (Waters Delta Prep 4000, Waters, Milford, CT, USA). The reversed-phase column was washed with 95% acetonitrile containing 0.1% TFA at a flow rate of 3.0 mL/min. The resulting peptide was further purified by semi-preparative reversed-phase HPLC using a 10 × 250 mm Kromasil C18 column. After lyophilization, the purity of the peptide was assessed by analytical reversed-phase HPLC using an Agilent Eclipse Plus C_18_ column (5 μm, 100 Å, 4.6 × 250 mm) with a 25 min linear gradient of 10–50% buffer B (0.1% TFA in acetonitrile) at a flow rate of 1 mL/min. The purity of all final products was ≥95%. The concentration of the peptides was determined by UV absorbance and a theoretically calculated molar extinction coefficient ε (280 nm) of 1490 mol/L^−1^ cm^−1^ based on the number of tyrosine (Tyr) residues (all peptides tested contain Tyr) [40].

The GI and MI variants that could not correctly form from one-step folding were synthesized with a two-step method described below (determination of disulfide bond connectivity).

### 4.3. Determination of Disulfide Bond Connectivity

The disulfide connectivity of the one-step oxidative-folding products of GI and MI variants was determined as described previously [41,42]. Briefly, linear peptides containing an acetamidomethyl (Acm)-protecting group at the C2–C4 or C1–C4 position were synthesized and then folded by incubation in 0.1 M NH_4_HCO_3_ (pH 8.0) at room temperature for 24–72 h. The folded products were further oxidized with an iodine mixture containing 30% CH_3_CN/2% TFA/68% H_2_O for 10 min to yield peptides with–S–S– bridges at “C1–C3, C2–C4” or “C1–C4, C2–C3”. The mixture of this second oxidized product and the one-step folding product of GI or MI variants was analyzed by HPLC: one peak represented a one-step folding product possessing the known disulfide connectivity.

### 4.4. Circular Dichroism (CD) Spectra

A Chirasscan plus spectrometer (Applied Optophysics, London, UK) was used to determine the secondary structure of the GI and MI variants. Each toxin was diluted with 10 mM phosphate buffer (pH = 7.2) to a concentration of 35 µM. The test conditions were: wavelength of 190–260 nm, scanning rate of 60 nm/s, sample cell path length of 1 mm, 3 repeats.

### 4.5. Oocyte Two-Electrode Voltage Clamp

Rat muscular nAChRs α1[L9S] [43], β1, δ, and ε plasmids were purchased from Addgene. The α1 plasmid was corrected to normal α1 by PCR. The expression of nAChR subunits in *Xenopus* oocyte and electrophysiological tests were performed as described previously [18,36,44]. Briefly, each oocyte was injected with 45–55 ng cRNA (1000 ng/µL) of α1, β1, δ, and ε at a ratio of 2:1:1:1. Then it was incubated in ND96 solution (96.0 mM NaCl, 2.0 mM KCl, 1.8 mM CaCl_2_, 1.0 mM MgCl_2_, and 5 mM HEPES; pH 7.1–7.3) containing 2.5 mM pyruvic acid sodium, 0.1 mg/mL BSA, and antibiotics (10 U/mL penicillin, 10 µg/mL streptomycin) (Gibco by Life Technologies, Grand Island, NY, USA) at 18 °C. Electrophysiological recordings were performed 2–5 days post-injection at 22 °C. The oocyte was exposed to the ACh pulse for 3 s every 5 min; the concentration of ACh was 200 µM. The membrane potential was clamped at −70 mV, and the ACh-gated currents were recorded with a two-electrode voltage-clamp amplifier Axoclamp 900A (Axon Instruments Inc., UnionCity, CA, USA). In high-dose experiments (1 μM or greater), 5.5 μL of a 10-fold concentrated conotoxin solution was directly pipetted into a static bath 5 min before the ACh pulse was exposed.

In the on-rate and off-rate kinetics for GI, MI, and their variants’ inhibition of the muscular receptor subtype, the gravity-driven perfusion of oocytes with ND96A was performed at a rate of 2 mL/min. Once a stable baseline was achieved, the cells were continuously perfused with conotoxin dissolved in ND96 solution and pulsed with ACh for 3 s at 2 min intervals. The association and dissociation rate constants were calculated from a single-exponential equation (Y = Y_max_ × (exp(−*k_off_* × t)) for dissociation and from the equation Y = Y_max_ × (1 − exp(−k_obs_ × t) for association, where Y_max_ is the bound ligand at equilibrium. The dose–response data were fit to the equation: % response = 100/[1 + ([toxin]/IC_50_) n], where n is the Hill coefficient, and IC_50_ is the antagonist concentration giving half-maximal response, by nonlinear regression analysis using GraphPad Prism (GraphPad Software, San Diego, CA, USA).

### 4.6. Toxicity Tests

Mice were randomly divided into groups treated with the GI or MI variants and a control group treated with wild-type GI or MI; 10 mice were included in each group, half of them males, and half of them females. We intraperitoneally injected into the mice 200 µL of saline solution containing different amounts of toxins. The time to death of each mouse and the number of deaths were recorded in 24 h. The time to death was expressed as the average value ± standard deviation.

## Figures and Tables

**Table 1:** Amino acid sequence and inhibitory activities of GI and MI and their variants with respect to muscular nAChRs.

NO.	Name	Amino Acid Sequences	Theoretical MW (Experimental) (Da)	IC_50_ (nM) (95%) Confidence Intervals
	GI	ECCNPACGRHYSC *	1436.52 (1437.49)	42.0 (38.8–45.4)
1	GI[∆8G]-I ^a^	ECCNPACRHYSC *	1379.49 (1380.46)	248.1 (221.3–275.9)
2	GI[∆8G]-II ^b^	ECCNPACRHYSC *	1379.49 (1380.41)	50.4 (45.9–55.4)
3	GI[+13G]	ECCNPACGRHYS***G***C *	1493.54 (1493.51)	>200
4	GI[+13A]	ECCNPACGRHYS***A***C *	1507.55 (1508.47)	45.4 (41.7–49.3)
5	GI[+13R]	ECCNPACGRHYS***R***C *	1592.62(1593.51)	73.5 (69.2–78.1)
6	GI[+13K]	ECCNPACGRHYS***K***C *	1564.61 (1565.50)	47.7 (44.2–51.5)
7	GI[+13G, +14G]	ECCNPACGRHYS***GG***C *	1550.56 (1551.48)	>200
8	GI[+13K, +14A]	ECCNPACGRHYS***KA***C *	1635.65 (1636.54)	>500
9	GI[+13A, +14K]	ECCNPACGRHYS***AK***C *	1635.65 (1636.54)	>500
	MI	GRCCHPACGKNYSC *	1492.59 (1439.38)	7.0 (5.6–8.8)
10	MI[∆9G]	GRCCHPACKNYSC *	1435.57 (1436.46)	>500
11	MI[+14A]	GRCCHPACGKNYS***A***C *	1563.63 (1564.52)	>200
12	MI[+14R]	GRCCHPACGKNYS***R***C *	1648.69 (1649.58)	22.2 (19.8–25.1)
13	MI[+14K]	GRCCHPACGKNYS***K***C *	1620.68 (1621.58)	62.7 (57.9–67.9)
14	MI[+14K, +15A]	GRCCHPACGKNYS***KA***C *	1691.72 (1692.61)	>500
15	MI[+14R, +15A]	GRCCHPACGKNYS***RA***C *	1719.73 (1720.62)	>500

^a^, GI[∆8G]-I contains a disulfide bridge “1–3, 2–4”; ^b^, GI[∆8G]-II contains a disulfide bridge “1–4, 2–3”; *, C-terminus is amidated.

**Table 2:** Kinetic analysis of the onset and recovery from inhibition of muscular nAChRs.

α-CTX	*k*_off_	*t*_1/2_^a^	*k*_obs_^b^	*k*_on_	*K*_i_^c^
	min^−1^	min	min^−1^	min^−1^ M^−1^	M^−9^
GI	0.16 ± 0.03	4.26 (3.16–6.54)	0.49 ± 0.02	0.33 × 10^7^	48.48
GI[∆8G]-II	0.10 ± 0.01	6.63 (5.25–8.99)	0.18 ± 0.01	0.08 × 10^7^	125.0
GI[+13A]	0.10 ± 0.02	7.04 (5.26–10.65)	0.23 ± 0.02	0.13 × 10^7^	76.92
MI	0.07 ± 0.01	9.82 (7.67–13.67)	1.92 ± 0.31	1.85 ×10^7^	3.78
MI[+14R]	0.18 ± 0.02	3.78 (3.12–4.80)	1.40 ± 0.31	1.22 ×10^7^	14.75

^a^: *t*_1/2_ = 0.693/*k_o_*_ff_; ^b^: *k_obs_* = *k_on_*[toxin] +*k_off_*; ^c^: *Ki* = *k_off_*/*k_on_*; *t*_1/2_ is the time required for a 50% dissociation of conotoxins from muscular nAChRs, *k_on_* is the association rate constant, *Ki* is the inhibition constant; data are means ± SEM from experiments with five–eight oocytes. Numbers in parentheses indicate the 95% confidence intervals.

**Table 3:** Toxicity of α-conotoxins GI and MI variants in mice.

α-Conotoxins GI and MI Variant	20 µg/kg		40 µg/kg		80 µg/kg		120 µg/kg		160 µg/kg		200 µg/kg	
	Death Time (s)	Death Rate (%)	Death Time (s)	Death Rate (%)	Death Time (s)	Death Rate (%)	Death Time (s)	Death Rate (%)	Death Time (s)	Death Rate (%)	Death Time (s)	Death Rate (%)
GI		0	1075.3 ± 150.5	100								
GI[Δ8G]-II					0	0	1462.8 ± 851.9	60	886.6 ± 392.0	80		
GI[+13G]							1036.6 ± 215.6	30			610.5 ± 239.6	90
GI[+13A]							0	0			1356.1 ± 41.1	20
GI[+13R]			0	0	1441.5 ± 231.0	80	929.2 ± 297.3	90	704.5 ± 57.6	100		
GI[+13K]			1619	10	1199.9 ± 383.4	90	771.3 ± 96.8	100				
GI[+13G, +14G]											0	0
GI[+13K, +14A]											0	0
GI[+13A, +14K]					0	0	0	0			1107.2 ± 250.3	100
MI	1185.7 ± 167.5	100										
MI[Δ9G]											0	0
MI[+14A]											0	0
MI[+14R]											0	0
MI[+14K]							0	0			895.5 ± 383.9	20
MI[+14K, +15A]											0	0
MI[+14R, +15A]											0	0
